# Foreign Psychological Literature

**Published:** 1858-10-01

**Authors:** 


### FOREIGN PSYCHOLOGICAL LITERATURE.

Our quarterly *resumé* of Foreign Psychological Literature will embrace an analysis of the following interesting subjects:—

1. On the Physical Symptoms of Insanity.
2. On the Responsibility of the Deaf and Dumb.
3. Singular Case of Insanity. Cysticerci in the Brain.
4. Pathological Anatomy of the Obliteration and Aberration of the Relative Functions.
5. Case of Sporadic Cretinism.
6. Somnambulism and extraordinary Neuroses generally.
7. On Disordered Sentiments and Affections.
8. Case of Mania with Homicidal Tendency.
9. On the Pathogenic Influence of Loss of Sleep.

*On the Physical Symptoms of Insanity.* By M. Sauze.

M. Sauze is of opinion that the physical symptoms of mental aberration have been much neglected, and agrees with M. Aubanel that if we could always observe the patient accurately, or if we could receive a trustworthy account from him of all his sensations, we should generally find irrefragable proof that *some organic change* is taking place in the brain of the person about to become insane. "Some *alienistes* only see in insanity an intellectual disorder independent of any material lesion. This is a grave error, which must lead to serious consequences. It would lead us to neglect the physical signs which *constantly* precede, and which accompany the outbreak of different mental affections; and which are the source of most valuable indications. Insanity is not a malady independent of organization. Although, as M. Leuret says, we are completely ignorant of the nature of the cerebral alteration, it is not the less true that this alteration does exist. Between the cause which produces the derangement, and its actual outbreak, there passes a series of phenomena, *constant and invariable*, which indicate with certainty that the organization participates in the production of the mental phenomena. We have never seen these physical signs wanting."

Before the delirium commences, there exists physical derangement, the importance of which is often overlooked. There is headache, and spinal sensations, such as of compressing or *tearing* the head—of emptiness or cold in the cranium. At the same time there is obstinate insomnia, which gradually undermines the strength. The digestive functions present noteworthy irregularities,—there is loss of appetite and constipation—the tongue is white and thickly furred. The general sensibility is modified by vague pains in the limbs; the mobility by spasms and tremblings: at the same time the intellectual and moral nature continues intact. "If you add to these symptoms, the flushing and heat of face, and the injection of the eyes, you can only see in this array of physical phenomena the symptomatic expression of a pathological change in the brain." Sometimes there is a true febrile action. The skin is hot, the pulse frequent and full, the tongue white, and the limbs painful; and the condition is mistaken for that of ephemeral fever.

At other times we may notice a state of dulness or somnolence which precedes the outbreak of the delirium. The patients have not their ordinary activity; they sleep often without any fatigue to account for the tendency. Sometimes they complain of heat in the head, and experience momentary relief from cold and wet applications. (An illustrative case is given in which these last symptoms continued for six months before the disorder of the intellect appeared.)

"This initial period of insanity, which has been designated as the *period of incubation*, is *constant*. It is observed generally on the *debût* of monomania, of mania, of paralytic insanity, and of stupidity or dementia: the physical symptoms are the same; the duration variable, from a few days to several months." These symptoms, likewise, continue after the actual accession of the mental disorder; but if overlooked before, they are still more likely to be so now, when other phenomena of so marked a character have appeared. "If, however, we cannot always trace them, we must not too hastily conclude that they do not exist; they are often only marked by agitation and delirious ideas; and whenever calmness and lucidity supervene, we can always detect incontestable traces of the physical disorder." It is not infrequent to observe at the cessation of the delirium, the same train of physical symptoms as those which preceded and attended the outbreak. The case given as illustrative of this position is interesting. A female, aged fifty-two, had disturbed sleep, and symptoms of lumbago; also headache, excessive weariness, fever, and gastric derangement. In about a month agitation and delirium supervened. She was subject to continual fears, heard divers noises, and voices which said unpleasant things. The treatment was exclusively physical, and lasted three months; the cure was complete; but at the decline of the malady, when the delirium disappeared, the affections of the head and stomach reappeared for a certain period.

"Experience tells me that a cure is not to be looked upon as solid and durable until these remaining physical symptoms disappear. Baths in the evening, purgatives, and local evacuations of blood, have given me the best results in these cases." M. Sauze views these bodily symptoms as more characteristic of the disease than even the mental disorder; for he observes that these latter are the first to disappear, and the last to be developed. These symptoms are clearly the expression of a cerebral affection, and appear to bespeak a "sort of state of irritation of the encephalon, a congestive or subinflammatory condition. Not that I would deduce from this necessarily the desirableness of a strictly antiphlogistic treatment in all cases; yet I think, judging from the results of my practice, that at the period of the *debût* of the affection, some few applications of leeches to the anus or the mastoid regions, with purgatives and revulsives, will often be followed by favourable results." These remarks are not, however, to be applied to all cases; there are those in which the physical signs are those of depression and an anæmic, or cachectic state; then the treatment must be completely changed, and we must depend upon good nutriment, with iron and other tonics.

The conclusions of M. Sauze are as follows:—

1. Insanity is a cerebral affection, characterized by headache, sleeplessness, disorder of the general sensibility and the digestive functions, and by trouble of the intelligence.

2. The two orders of symptoms, physical and moral, are *equally* indispensable to characterize insanity. Every definition which would exclude either, would be incomplete, inexact, and would give a false idea of the malady.

3. Until our own times, insanity has scarcely been studied in any save its intellectual aspect: the physical symptoms have been mistaken and neglected.

4. The physical symptoms are especially manifest in the commencement; but they are manifest both during the course and in the decline. They always precede for some time the actual outbreak.

5. Insanity being a cerebral affection in all respects like any other organic malady, requires in like manner physical treatment.

6. This treatment ought to be applied *at the beginning. At this epoch insanity is almost always curable.*

7. Moral treatment must be considered only as an adjuvant—as the hygiene of the brain.

8. Insanity only becomes incurable because the proper physical treatment is neglected at the proper time.

9. The first origin of insanity is often connected with the various forms of degeneration of the human race, and with the existence of neuroses.

10. It is not sufficient to study only the treatment of insanity in its actual state. It is especially necessary to study the means of preventing it, and to establish the laws of prophylaxis.

[These views are highly important, both in themselves, and as emanating from such high authority. Yet we can scarcely accord that the physical aspect of insanity has been so entirely overlooked as is here presupposed, nor that this branch of the treatment has been so neglected. Recognising the necessity for the strictest attention to the physical treatment of the disease, we must not overlook, nor put too much in the background, that of a moral nature. Granting even that the bodily functions were *always* disordered, it cannot be forgotten that in many instances the affection is induced, proximately at least, by moral influences; and the force which has been powerful for evil may in many cases be counteracted, and its effects neutralized by the same order of forces, otherwise applied.]

---

*On the Responsibility and Legal Relations of the Deaf and Dumb.* By Dr. Jendritza. (Zeitschrift, Jan. 1858.)

S——, born deaf and dumb, now twenty years of age, of powerful constitution, was brought up without any education or instruction by his parents, poor country people, who supported themselves by day-labouring, and was from early youth made useful in many branches of their arduous occupation. As his bodily powers seemed completely developed when about seventeen years of age, he was fully employed then by his father in heavy work. After two years and a half of this, the father died, and the widow, fearing that she should not be able to support herself, only aided by the deaf and dumb son, took a situation which then offered itself; whilst a distant relative, N——, who kept a small inn in the country, became guardian to the son, and took him to assist in the hostelry. After some time N—— had much reason to be dissatisfied with him, partly on account of extreme laziness and awkwardness, which showed itself in many duties; and, on the other hand, on account of sudden ebullitions of anger after reproof or punishment, during which he would throw down anything with which he might be engaged, and run away.

On the 2nd of November, S—— was engaged in harnessing the horses to the wagon. One of the horses had only been for a short time so employed, and was every day very awkward about the harnessing and resisted the attempts of S——, by jumping about, and lashing out his heels, so that S——'s wrath was roused, and he was soon engaged in a formal contest with the horse, and struck him violently and repeatedly. N——, hearing the uproar, looked out of his window, and was very angry on seeing the horse so ill-treated. He seized a stick, and rushing upon S——, he pulled him away, and struck him again and again. S——, still in the greatest excitement about the horse, was still further exasperated by the unexpected punishment. Smarting from the blows on his back, he turned to the wagon, and seizing one of the bars, he flew towards N——, threatening him with it. He, perceiving the intention of S——, had taken a few steps in retreat, calling loudly for help. But S—— speedily overtook him, and striking him on the head with the iron-bound bar, smashed in the skull, so that N—— fell dead at once, without a cry. Upon the call for help being heard, the maid-servant hastened to the scene, but only arrived in time to witness the completion of the murder; she saw the blow struck, and N—— fall. S—— threw away the bar, and fled across the fields. Examining more closely, the servant convinced herself that N—— was dead. Called together by her outcries, the neighbours arrived, and being informed of what had happened, they pursued S——, and soon brought him back and bound him. He was on the same day committed to legal custody.

On the first hearing he was convicted of the murder through the testimony of the girl, who understood how to communicate with him by signs, and informed him that she had been a witness of the blow. Afterwards his mother was employed as interpreter by the judge; and after speaking with him in his manner, alleged on his behalf, that for a long time he had been very much irritated against N—— by often repeated punishment; but immediately before the fatal blow, having been roused to fury by the unmanageable horse, he had been driven to perfectly uncontrollable rage by the severe punishment that followed, and wished at last to revenge a long series of injuries; but added that he did not intend to kill N——. Hereupon the judge requested an opinion as to the responsibility of the accused, which here follows:—

The deaf and dumb man, S——, cannot be viewed as a responsible person in respect of the manslaughter of the innkeeper N——, on account of being born deaf and dumb, and as the evidence shows, from not having received the least instruction or education.

In justification of this view, it is necessary to bear in mind the distinction between those deaf and dumb who are born without hearing, or lost it in early infancy before they had learned to speak; and those who had once possessed those faculties, and had lost them after the reception of some education. The former are truly the deaf-mutes; the latter may be called simply deaf and dumb. *These* have some responsibility in the eye of the law; but the former, inasmuch as for the comprehension of social and civil relations hearing is necessary, remain ever irresponsible. Such of these as have received no education, as is the case with S——, lack the faculty of communicating or exchanging ideas, except through the medium of certain signs. Even those who have received some degree of education must still be considered as only endowed with a limited and relative responsibility; as, through the failure of hearing and speech, the development of the mind and soul must necessarily be imperfect.

This opinion is founded on the observation, that even in the best educated and most talented among the deaf-mutes, during their whole life traces remain of their former early condition, which are outwardly manifested by certain abnormal physical manifestations; and which remind the careful observer of conditions which were obvious at the earlier periods, before education commenced. . . . . . Under the most complete system of education of the deaf and dumb, we must yet doubt whether they can arrive at any deep practical comprehension of right, obligation, duty, necessity, and ideas of the like nature. . . . . . Again, all deaf-mutes, however educated, retain a persistent proclivity to sudden accessions of rage which are sometimes uncontrollable. Also all their passions and appetites are more strongly developed than in those of sound senses. Control of emotions and instinctive propensities cannot be taught in the same full and practical manner that the knowledge of physical objects, and even arts and sciences may be conveyed. . . . . . For these and other reasons, the deaf and dumb cannot be compared as to responsibility with those of perfect senses; nor can they ever merit the full punishment of crime, the justice of which should involve a comprehension of moral relations of right and wrong, much deeper than that of the dog which fears and flees from the whip, and which it may still remain a matter of doubt whether the deaf and dumb comprehend.

Whilst the educated deaf-mutes only possess a relative and limited responsibility, those who, like S——, are utterly without any instruction, possess none whatever, and are merely in a state of nature. Such a man is of the lowest grade of humanity, and can be held as little higher than the beasts which perish. Without any consciousness except that of his natural inclinations, he wanders amongst men, incapable of communicating with them, and cannot, from sensuality, raise himself up to any idea of virtue.

S——, roused to anger by long ill-treatment, and immediately by the horse, and the punishment succeeding, committed an act which would be murder in one endowed with perfect senses. He thought not of the consequences nor of the meaning of the deed, but only obeyed the blind impulse to revenge his injuries. In the eye of science and of the law, he must be considered as irresponsible. His removal to an asylum for deaf-mutes is advised, in order to his receiving some education; and afterwards it is recommended that, to avoid future evil, he should be placed under competent surveillance.

---

*Singular Case of Insanity. Cysticerci in the Brain.* By Dr. Czermack. (From the *Correspondenz-Blatt.*)

Marie K——l, born in 1818, the third child of healthy parents, bodily and mentally normally developed, was in the condition of a useful servant for house and field work. She menstruated regularly from her twelfth year. She was married at the age of twenty, and had three sons.

In the autumn of 1853, she complained of a violent pain at the crown of the head, and dragging and tearing pains in the left side of the face, and in the left arm and shoulder. There was no visible change in the painful localities. This affection was treated by various physicians for rheumatism, but without effect. The pains continued more or less violent for more than a year. She was during this time for the most part confined to her room and bed, and could not follow her usual avocations.

She was patient and composed during the most excruciating pains; but was sullen, impatient, and peevish *when the pains were relieved.* By degrees she complained less of pain, but grieved to be so long disabled; the consciousness of the necessity of looking after the house was a trouble to her. The gloomy aspect increased, and *the pains by little and little vanished entirely.* She withdrew from the family, would allow no one to trouble themselves about her, and took little nourishment—at times, none; if urged, she would grow angry.

She began to walk rapidly up and down her apartment, and to talk aloud to herself, gesticulating energetically. She began to complain of having brought all kinds of evil upon her family, and *upon the world at large;* she was the vilest of creatures, not fit to live. Her nights were for the most part sleepless.

One day she left her chamber and threw herself into some neighbouring water, but was taken out by her sons. She was then quiet a few hours, after which she flew at one of them, saying that she must kill him, that she herself might die. On this day, two years after the commencement of the pain, Dr. Czermack first saw her. (Here follows a description of her *personnel* and general aspect). The tongue was loaded, the respiration quickened, the pulse 120, and the general heat increased; food and drink were refused, the bowels were confined, but urine passed copiously. She took no notice of any questions, but screamed and talked incoherently. There often seemed to be a tendency to *rhyme* and *rhythm* in her expressions. She fought and bit constantly, and sleep was entirely absent. After six days these prominent symptoms disappeared; emaciation increased, the muscular system relaxed, the expression of the countenance became heavy and immovable, the eyes half closed, the pupils dilated, the reaction of the iris slow, the glance feeble and anxious, the tongue dry, the thirst increased, the pulse 70, the temperature of the body was diminished, the bowels confined, and the urine scanty. She complained of faintness and occasional vertigo; she felt very sad and depressed, and had a confused idea that she was "changed into another person."

She sat shrunk together in the darkest corner of the chamber; she trembled over the whole body if any one approached or spoke to her. She answered with feeble voice only such questions as related to her own condition, and took no notice of any other observations. Her movements were slow and uncertain. She only took food after much persuasion, but allowed herself readily to be washed and dressed.

In the course of about ten weeks, the countenance became smiling, but the glance staring; the right pupil was much dilated, and did not answer to the stimulus of light; the left pupil was contracted, and did not dilate when light was withdrawn. The fibrillæ of the muscles of the body, face, and tongue trembled. The pulse was 96, the temperature of the head slightly raised; the emaciation was increased.

She remained in a corner of the chamber, and played with toys or pieces of wood; she replied to questions by a laugh, swallowed greedily her food, and attempted to make her wants known by loud inarticulate sounds. Her gait was uncertain, and she dragged the right foot slightly. She was indifferent to surrounding circumstances, and neglected cleanliness entirely.

This condition lasted two years, after which she died of pneumonia. These are the principal morbid changes found on examination forty-eight hours after death:—

The dura mater was tightly stretched, very much injected, and between it and the inner membranes was a large quantity of yellowish transparent serum. The inner membranes were dull and milky over the whole hemispheres, containing fluid, thickened, here and there injected, and easily separated from the substance of the brain.

On the inner surface of the pia mater, and between the convolutions were found fifty-seven clear roundish vesicles, twenty-nine as large as millet seeds, seventeen as large as peas, and eleven as large as beans. Each of these vesicles contained a worm-like entozoon (Blasenschwantzwurm). Most of these were on the right side, but some also on the left. The greater number by far were on the middle lobe. On this point some of the concluding observations of Dr. Czermack are noteworthy:—

"As, according to Professor Huschke, the central convolutions constitute the 'point of indifference' (Indifferenz Punkt) of the brain, and may, physiologically, be viewed as the 'plastic expression of the soul' (für den plastischen Ausdruck der Seele), and of self-consciousness; so we must regard this part of the brain as that especially affected in all cases of mental derangement. In all such cases, where the consciousness and personal disposition, and the sense of individuality (the I) is affected, there are always morbid changes in this locality. This observation has been confirmed by me, in conjunction with Director Köstl, by examinations made on many hundred cases of insanity, and I add it to the comments on the previous case, without going further into the detailed arguments."

---

### *On the Pathological Anatomy of the Obliteration and Aberration of the Relative Functions.* By M. Follet, M.D.

M. Follet, in the outset, points out forcibly the necessity for analysing well the morbid changes found in the brain, and the errors committed in too hastily concluding that these changes are the *cause* of the foregoing phenomena.

"After having demanded of these examinations what there may be palpable, tending to explain the derangement of the intelligence, we shall show that the alterations remarked in the membranes, or the substance of the brain, appear to us not as the first cause of the *discord* produced in the instrument, but as an effect of the degeneration consecutive to those morbid modifications which, in a latent and gradual manner, have troubled the equilibrium of the hemispheres as to their innervation. It is time that this principle be recognised, and that the observer, because he has found here an inflamed membrane and there a softened patch, be not reduced to suppose that there is nothing beyond that; and, taking the effect for the cause, pass in silence the nervous disorder (*trouble*), which has from the beginning dominated over the vital powers. If, like the unknown quantity in a problem, this trouble is not in itself recognisable, it assuredly is so through the secondary alterations, which pass from the blood to the viscera, and affect in a tertiary manner the nutritive and assimilative functions, and so produce those morbid changes which, instead of being viewed as a result, are so often considered the primary cause of mental affections."

M. Follet weighs and measures each brain. In weighing, the cerebellum, the cerebrum, and each hemisphere are weighed separately. From this mode of examination, it has been shown that epilepsy coincides, with a difference of weight between the two hemispheres, the equality of which varies but little in the other types of alienation. In measuring, the thickness of the white matter, from the bottom of the convolutions to the surface of the ventricles, and the chord of the ventricular arch, are always taken—the latter measurement, in order to appreciate the dilatation of the ventricles. This has also brought to light an interesting fact—viz., that the thinning of the white substance is proportional to the degree of dilatation; and that this is a *vice of structure* inherent to congenital obliteration of the faculties, the conditions of which are more or less reproduced in all the forms of *acquired* obliteration. M. Follet considers the loss of equilibrium between the hemispheres as the point of departure of all mental pathology.

As to the cerebellum, "an organ quite independent of the cerebrum," we remark that, in middle age, its weight is correlative with the height of the subject; before twenty years of age, it is proportionally greater; after seventy years, much less.

M. Follet considers the alterations in the cranium and membranes to be produced in proportion as

"Under the membranes there is condensed an excess of *caloricity*, by the abnormal disengagement of that nervous influx, of which the rupture of the equilibrium between the hemispheres will be, sooner or later, admitted as the latent cause and primordial element in all mental affections. And by reason of the *solidarity* between the nervous and the vascular systems, it will be recognised that this inter-hemispheric disturbance, after a period of incubation, reacts upon the blood, which thence becomes elevated in temperature, and, acting upon the membranes, constitutes a state of *cerebral febricity*."

An action is also instituted upon the vessels themselves, sometimes obliterating them; and thence results the softening of the subjacent tissue. Much of M. Follet's analysis of the changes of the membranes (in which nothing particularly new appears) is devoted to proving that those changes are only secondary.

On the alterations in the brain-substance, M. Follet holds the same opinions:—

"Having shown that the changes in the membranes are an *effect*, not *cause* of the mental alienation, we add that the same is the case as to the brain itself. It is in the inner structure of the brain, serving for the manifestations of the soul, in the secret and mysterious play of that nervous influx, in the disturbance of its equilibrium, that we must look for the essential cause of the trouble which grows and progresses in the relative life. The nervous and sanguine elements are so intimately connected, that if the first *oscillates*, the second participates in the febrile state; and it is only after a series of actions and reactions between these two powers, that first the pia mater, and afterwards the other membranes, become affected; and that, under the influence of this physical aggravation, atrophy and softening of the brain substance takes place."

M. Follet has no hesitation in considering the white matter of the brain as the seat or organ of attention and memory; and its thickness varies inversely as the dilatation of the lateral ventricles, as measured by the chord of their arc.

The general conclusions from several hundred examinations are as follows:—

1. Obliteration of the relative faculties is incurable, whether congenital (idiocy) or acquired (dementia). General or partial aberration is susceptible of cure, if treated in time.

2. All subjects that have preserved any intellectual aptitude, have presented a thickness of white matter of from 10 to 15 millimetres (about $\frac{1}{3}$ to $\frac{1}{2}$ an inch), and the chord of the ventricular arc has presented a medium of about 10 centimetres (nearly $1\frac{1}{3}$ inch). During life we have the exact condition of idiots, imbeciles, and the demented, defined by the amount of attention and memory; and these psychopathic lesions are in strict relation to the thinness of the white matter and the dilatation of the ventricles. There is always by so much the more loss of memory and general intellectual enfeeblement, as the thickness of the white matter declines below 5 millimetres and the ventricular chord exceeds 10 centimetres. These observations bring us to the conclusion that the attention, which is the generative principle of the ideas, and the memory, are inherent to the white matter, which seems to us not to be a simple conducting substance, but the centre of reflection and the common seat of memory—the source of our intuitive impressions, whence arise our metaphysical ideas.

3. The absolute weight of the encephalic mass does not correspond with intellectual power or adaptability. Were it so, the idiot would sometimes take first rank. The structural conditions with which intellect seems to correspond are—

A. The development of the anterior lobes.
B. The symmetry of the convolutions, giving extended surface to the cortical matter, the seat of sensibility, of intelligence, and of will.
C. The thickness of the white matter—the seat of memory.
D. The narrowness of the lateral ventricles.
E. The equality of structure and of weight in the cerebral hemispheres, conducing to the equilibrium of their innervation.

4. The cause, the march, the complications, and the degeneration of mental aberration may be thus formulated:—

A. Interhemispheric nervous disturbance of equilibrium (from moral or physical causes) acting at first in a latent manner. At this period peculiarities of manner, character, or actions show themselves.

B. A febrile state, consecutive to this breach of equilibrium. This is the period of illusions, hallucinations, and increase of delirium.

C. An inflammatory condition of the pia mater, frequently terminating in death.

D. A chronic form, in which the capillaries of the pia mater are obliterated, and the brain-tissue begins to be atrophied.

E. There may arise a corresponding pathological condition of thorax or abdomen, which dissolves the vital unity.

F. The cerebral innervation is lowered, relative life is obliterated, and physical life is extinguished in general progressive paralysis.

5. Epileptic attacks repeated often in the day may correspond with a difference in the weight of the hemispheres to the extent of 200 grammes (nearly 7 ounces). These frightful attacks are due to a loss of equilibrium in the innervation of the hemispheres. If the attacks are very distant from each other, there is occasionally great danger of a catastrophe. We have just lost an epileptic patient from apoplexy, who for six months had appeared to be in the way of recovery.

M. Follet indulges in a little theory, upon which, however, he does not dwell, that the grey matter is but the superficial expansion of the white substance, tinged by the constant contact of the nervous fluid, which is, as it were, insulated under the membranes.

The practical suggestions resolve themselves into one or two very important points. Idiocy and dementia are *incurable;* but there is reason to hope that the acute aberrations, whether continued or intermittent, may in time be as amenable to successful treatment as any other diseases, provided always, and above all other considerations, that appropriate treatment be adopted *at a sufficiently early period.* M. Follet speaks most highly of influencing the blood through the *ingesta,* and so acting indirectly upon the innervation. Temperature also has a mighty influence in calming excitement amongst the direct modes of acting upon the innervation. M. Follet speaks most favourably of the pyrophosphate of iron and soda, which, he says, appears to act most powerfully upon the blood and the nervous element.

---

### *Case of Sporadic Cretinism.*

M. Baillarger introduced during the year 1857 to the *Société Medico-Psychologique* a cretinous girl, with remarks, from which the following are abstracted:—

"You know, Messieurs, the differences which I have held to be established between cretinism and idiocy. Cretinism is, according to my views, an arrest of development of the entire organism. Endemic idiocy is characterized, on the contrary, by an arrest of development of the intelligence, which establishes a well-marked difference between these affections. I have just met with a very remarkable case of sporadic cretinism:—It is a girl, born at Melun, of healthy and well-formed parents. She presented no peculiarity at birth; she began to walk at fifteen months, and at this epoch she had not a more voluminous head than children at that age generally, nor any other distinctive marks. The first dentition was completed at three years of age, and it was then that the general development was arrested.

"The girl is now about twenty-seven, and she has the intelligence and tastes of a child of four or five; she plays with a doll, and has no sentiments of modesty. It is in vain to attempt any education, she could never learn to read, and can scarcely count twenty. Speech is easy and pronunciation clear, but the voice is nasal. Her height is about three feet, the head is elongated and flattened at the sides, the palatine arch is high and prolonged, the tongue is thick. The features recal in all points those which we assign to cretinism; the nose is flat, the mouth large, and the lips thick. The body is fat, the limbs thick and short, and sufficiently regular. The second dentition only commenced at eighteen years of age, and has not yet terminated. The pubis is smooth, the mammary glands rudimentary; menstruation has not been established, and there has never been any sexual sensation.

"It seems to me that, in an anthropologic point of view, there is much interest in studying those types which prove that in the human race there are beings that cannot attain complete development. But I wish to submit to the Society an idea which has occurred to me in connexion with this individual case. This girl presents an extraordinary polysarcia. I have before seen in Paris, with M. Rayer, a little girl who had the same formation; I have also remarked in the Pyrenees, the extreme obesity of certain cretins. I have considered whether there is not a relation between the general condition of these arrested organisms and that of those submitted early to castration. The description which M. Virey gives of eunuchs, is this:—'Softness, paleness, and flaccidity of the flesh; relaxation of the cellular tissue, great development of the lymphatic and glandular system, absence of hairy appendages, soft and relaxed abdomen, large thighs; the legs swelled, moisture predominating in all the tissues, appearing old and decrepid early, little heat of skin—hence the name of *frigidi* given to eunuchs.'

"In reference to this last fact I have one more relation to notice: the mother of this girl has informed me that she has a great tendency to coldness, and that in winter she has to leave her almost entirely in bed, on account of her being so cold. The picture which M. Virey draws of eunuchs, and what we see of cretinism, authorizes then some relation; and I believe that we may entertain the idea that the polysarcia is due to the absence of menstruation, and the inactivity generally of the generative organs."

M. Baillarger afterwards explained that this *only* related to the polysarcia, and not to the general condition of arrest of development—the polysarcia he believed to be a subsequent effect. Further, idiots and imbeciles have generally the sexual organs well-developed, and these have the reputation of being salacious. Idiocy and imbecility are characterized by arrest of the intelligence; but in cretins the generative organs are little developed, or are large, soft, flaccid, infiltrated, and without any kind of power—in a word, they are undeveloped or imperfect. As to the general state, it is due to a special cause, to which also must be referred the arrest of development of the genital organs, in the same manner as arrest of development of the stature, and of dentition, &c.

M. Ferrus, commenting on this case, did not in all points agree with M. Baillarger, not considering the portrait drawn by him represented truly the cretin type. He continued—

"M. Baillarger has expressed formally the opinion that the arrest of development in its essence attacks especially the generative apparatus, the organs of which are soft, flabby, infiltrated, and powerless. I consider that this apparatus possesses more power than is here attributed to it. I have seen cretins have powerful erections; and indeed it could not be otherwise, inasmuch as the great scourge of those countries where cretinism is rife, is that cretins beget cretins. Fodéré has fully shown that cretinism perpetuates itself in a great number of families. As to the simultaneous existence of idiots and cretins in the same locality, we cannot deny that where cretinism is endemic, there are idiots and imbeciles arrested in development, but here cretins are the rule and idiots the exception."

M. Morel agreed so far with M. Baillarger as to grant the accordance between eunuchs and cretins as stated by him, but guarded against the supposition that all those who are affected by sterility or infecundity, are related to either of these unfortunate classes. Many of them are incapable of propagating the species in the normal state, in accordance with the laws developed in the treatise on the "Degenerations of the Human Race." These degenerate beings are distinguished by short stature, bad formation of the head, and often of the thorax; incomplete or tardy development of puberty, and sometimes by complete inability to procreate. In spite of this latter fact, they are far from presenting the peculiar type of cretins or eunuchs. M. Morel believed that the characters of these degenerations are so marked, that the time will come when, by mere inspection of the subject, we shall be enabled to refer each form of degeneration to its specific cause.

---

### *Somnambulism and Extraordinary Neuroses generally.*

By M. Garnier and M. Ferrus.

On the 25th of May, 1857, a discussion took place before the *Société Medico-Psychologique*, in reference to a case recited by M. Cerise, and to "extraordinary neuroses" generally, which involved questions concerning somnambulism, natural and artificial, and magnetism. We purpose giving a full abstract of the proceedings of this meeting, to indicate the state of feeling of our continental brethren on these vexed questions. M. Garnier spoke first:—

"M. Cerise had related the experience of M. Puel on a patient affected with catalepsy. During the period of the accession, the patient at first suffered much from the application of the hand, but afterwards became accustomed to it; and then to friction, which, at first painful, became ultimately salutary. Frictions too difficult to repeat or prolong, were replaced by applications of cold water, which began by exasperating the patient, and ended by calming her. It seemed to result from these facts—that the remedy was effective as soon as the patient was accustomed to it, or had confidence in it. The experience of M. Cerise showed that the pointing of his cane towards an organ seemed to determine pain in the part. We might also recal the experience of M. Delasiauve upon a maniac who freed himself from his hallucinations, believing that he was thereby thwarting the machinations of his enemies. A general law seemed to result from these observations, common to all mental and nervous affections, that the conviction of the patient, what we call vaguely *imagination*, and what M. Delasiauve calls by the more precise name of *belief*, exercises a great influence on the condition of the patient, and determines, according to the opinion of the patient, the relief or the aggravation of the suffering."

From these observations, we are naturally conducted to somnambulism, in which it appears also that *belief* plays a great and important part.

The phenomena observed in artificial somnambulism are, partial or total insensibility, rigidity of the limbs, excitement of certain faculties, and, lastly, the transposition of the senses and vision through obstacles. Leaving aside for the moment this last phenomenon, the only one wonderful, we find the others presenting themselves to a certain extent in ordinary states, such as dreams, reveries, or natural somnambulism. Between these, then, and artificial somnambulism there is but a difference of degree. Thus, in profound reverie, a state so natural and common, the body is sometimes immovable, sometimes we walk slowly without perceiving surrounding objects; we are insensible to touch, or to voices that address us. On the other hand, the imagination is on fire: we represent vividly to ourselves the objects and persons on which our thoughts are engaged; we address words and gestures to them; we perceive about us only those things or persons that may have some relation with our internal workings. In reverie, then, we observe immobility, partial insensibility, and excitement of the faculties as regards certain objects or certain persons.

Dreaming presents the same symptoms. Sleep renders us in great measure immovable, and insensible to objects of perception. Notwithstanding which, the interior conception is active; it represents objects with such vivacity that they appear present; and the illusion is so strong, that we not unfrequently begin to talk, to move about, or even to get up and walk. If at such a time any one questions us in accordance with the subject of our dream, we perceive the words, and add our reflections and responses; being sensible thus to things relative to our dream, but insensible to all the rest. It is a partial waking state.

We come now to natural somnambulism, in which all the phenomena of dreaming and nightmare are more distinctly marked. The somnambulist is insensible to a great number of objects of perception, but very sensible to others. He walks upon the ridges of roofs, and does not perceive the abyss over which he steps; he answers to a feeble voice, which speaks to him on the subjects on which he is occupied, and hears nothing of the clashing of chairs and tables that are moved about to awaken him. Recal the case of Castelli, related in the "Encyclopédie:" he wrote by the light of a candle which alone he could see. If this were extinguished, and others lighted about him, he stopped writing, and went softly to relight his own. He had only perception for a certain number of things, and was profoundly insensible to all others. It was a state of partial waking. The opinion, and, so to speak, the intention of the somnambulist, determines the amount and *direction* of the perspicacity or lucidity: one intends to taste liquids, and perceives if they are changed; another is fixed upon drinking only one, and finds its flavour in pure water, substituted. It is true that the conviction or belief of the patient influences his perceptions, as in the examples of nervous and mental maladies before cited. One somnambulist has, as it were, taken his resolution not to awake, and he is insensible to pricking, burning, or pinching; another has his mind directed to awaking, and awakes by the touch of a feather.

This partial insensibility—this special perspicacity—conducts us to the phenomena of artificial somnambulism. The chief difference which separates this from natural somnambulism is, that the one is preceded by sleep, whilst the other is immediate. It takes place according to the conviction or belief of the patient. We do not think that there is any transmission of fluid from the operator to the subject, but only a belief on the part of the latter that he is about to fall into this state, which is sufficient to determine its occurrence.

The influence of *belief* on the production of this state was shown by the commission appointed by the Academy of Sciences about the close of the last century, of which Franklin, Bailly, and Lavoisier formed part. The phenomena of mesmerism were induced upon patients by *telling them* that they were subjected to the mesmeric manipulations; whilst none were produced by such performances, done *without the knowledge* of the subject. Since then, it has been asserted that it was not magnetism, but a fluid passing from the operator, that produced these phenomena; and that the results could be produced even in another room. When tried, it was said that the person operated on speedily began to manifest signs of some action upon her, to complain of heat, and remove her handkerchief, &c. But these signs are vague, and may be attributed to many other causes. In a crowded saloon, it is by no means unfrequent to find persons incommoded by the heat: perhaps many others had shown such indications, and had not been remarked. If the person in question had complained of cold, or any other sensation, it would no doubt have been attributed, in like manner, to the influence of the operator. When to one and the same cause we attribute such contradictory phenomena as heat and cold, we are very like to be deceived as to the true cause.

It is to this lack of precision in the relation of causes and effects, that we must attribute the one truly marvellous phenomenon of artificial somnambulism—that is to say, the pretended transposition of the senses, and vision across opaque obstacles.

Some have attempted to explain this latter on physical principles; as all bodies are porous, there might (it is said) pass through the most opaque medium a small quantity of light, imperceptible to eyes in the ordinary condition, but perceptible to the ecstatic regard of somnambulism. Thus all bodies would become transparent. But on this view, the bodies supposed to be seen ought to become also transparent, and therefore ought no more to be seen than the obstacles themselves.

It appears to us that the account given by somnambulists of the objects which they profess to see, is always sufficiently general and vague to apply to a great number of particular objects. A man, worthy of credence, told us that the first time he went to Marseilles he was taken to a somnambulist, who said to him, "You came from the North; your father lives in a small town,—I see him now; he is going into the cellar." On writing to his father, it was ascertained that at that time he was going into the cellar. We asked him particularly if the indication had been just so precise; if it had not been rather, "You are a stranger; your father is in a small space, and is going down" (*il descend*),—vague propositions which might coincide with a thousand circumstances. He could not deny that it might have been so, and would not affirm that the information was as precise as it appeared after the father's answer. It is thus that the Duc de St. Simon relating, after the death of Louis XIV., a pretended vision, anticipatory of the event, which a young girl, looking into a glass of water, had had, may have inserted into the prediction circumstances which he only knew by the event. An honourable member of this society has related that a somnambulist had told him that he had a button of gold and a twenty-five cent piece. But is he quite certain that he was told precisely the article, or only that he had *something* in his mouth, to which he unconsciously added the designation?

We cannot too rigorously guard against our taste for the marvellous; we love the supernatural, and through this love we often deceive ourselves. I have been present at a *séance*, where this disposition was shown in a striking manner, when the public refused to be undeceived by one who offered to undergo the same tests as the mesmerized person.

A final reason arrests me and prevents me believing in any vision through opaque obstacles. Somnambulists have never seen anything but objects without any interest either for themselves or others; they are always blind to objects which might be to them of very great importance. At the time of lotteries, the drawing of the winning numbers was made at Lyons, Bordeaux, Lille, and Strasbourg, about 10 A.M., and numbers might be purchased in Paris up to 11.30 A.M. It might have been very profitable to the somnambulists to see these numbers, but it has never been done. In our own days, after the landing of our troops in the Crimea, it would have been equal to a fortune to have followed our army, and seen the victory of the Alma at the moment. But those who made fortunes were by no means somnambulists. Those who say that the somnambulists are inspired by spirits, which also turn and make vocal tables, also say that these spirits have not any mission to make our fortunes. The spirits prefer stating how many chairs there are in the next apartment, and how many coins there are in our neighbour's purse. But those who do not believe in spirits, but only in transparent vision, should show us how it is that they only see frivolous things, and are blind to everything of importance.

In regard to those phenomena that are credible, there is but a difference of degree between them and those of reverie, dreaming, &c. As to the transposition of the senses and the translucent vision, these are explicable by considering the vagueness of the indications, some coincidences which have been noted, the neglect of failures, and, above all, our love for the marvellous.

*M. Ferrus* thought that, in order to unmask the dangers and the abuses of magnetism, it ought to be made a subject of special discussion before the society. Magnetism indeed has brought forth a host of juggleries, with which it would be well to deal scientifically. At a former epoch we have seen convulsionaires and mesmerists; in our own times we have table-turnings and familiar spirits. The highest saloons are open to skilful magnetizers; recently a lady was so vividly impressed by one of these exhibitions, that miscarriage was produced. We ought then to expose the phantasmagorias which impose upon so many of the weak and unreflecting, and also upon a few highly honourable persons. It is important, also, to trace the analogies of these phenomena in the pathological conditions occasionally met with. There is nothing completely mysterious and that may not be penetrated, at the root of these mystical phenomena. I beg to introduce two cases, which have reference to these questions.

Amid the infinite shades of variety which prevent any exact definitions, spontaneous somnambulism assumes two distinct forms; in the one, almost analogous or closely united to dreams, it appears a simple accident of ordinary sleep; in the other it approaches hysteria, catalepsy, and ecstacy. In the first it is very difficult, if not impossible, to enter into communication with the subject; and it would, perhaps, be dangerous to attempt it when in a perilous situation. In the second, on the contrary, where the attacks come on during the waking state, the subjects easily establish relations with their friends, or even with strangers. To these two conditions is allied a third—artificial magnetism, or induced magnetic sleep, in respect of which have arisen long, angry, obscure, and obstinate debates, which yet await a solution. We shall confine ourselves at present to a division of somnambulism into *nocturnal*, which takes place in natural sleep; and *diurnal*, which comes on in the waking state, amid the most diverse circumstances, and presents the most defined neuropathic characters.

The form assumed by my two observations is the latter; they both occurred in my own practice. The two subjects of them were young, recently married, happy women of society, and so far from having anything to gain by fraud, they had both the most powerful motives for concealing the affection, if it had been possible.

"Case I. Madame N. had been from her infancy subject to slight nervous affections, which were aggravated at the period of puberty. After this time they diminished, and only appeared at long intervals until just before her marriage. Of an hysterical character, these affections were then almost instantaneously developed under the influence of a strong moral emotion. She had witnessed an assassination. Marriage did not diminish, but rather increased their intensity; at a later period, their form was modified; from being hysterical, they were progressively transformed into a cataleptico-magnetic sleep, to which sometimes convulsive movements were added. From this condition she could not by any means be aroused, yet she answered exactly to the questions of her husband, who brought himself into relation with her by simple contact; and she predicted the return of the crisis, without preserving on her awaking the least remembrance of what had passed. Pathological conditions worthy of notice had preceded or coexisted with the somnambulistic condition; Madame N. had been almost completely deaf for two months, without obvious cause. This was now reproduced, apparently as a sequel to a jolt in a carriage, and lasted a year.

"There were frequent febrile attacks, with shivering and burning, during a space of fourteen months; and in this time her stature increased an inch, although she had passed the age of ordinary growth.

"The crises, which, as to duration, varied from one to two hours, were ordinarily preceded by uneasiness, agitation, numbness, and pandiculation; and immediately before the accession, an acute pain in the nape of the neck. MM. Lallemand and Planidoux, who were consulted, after some hesitation pronounced it a case of natural somnambulism.

"Madame N. was submitted at Nimes, where she then lived, with the consent of those two physicians, to the operations of a magnetiser, a relative. During the induced sleep, she gave marks of great lucidity.

"On another occasion, at Montpellier, when no one was present but her husband and M. Lallemand, who has often related the circumstances to me, Madame N., during the natural attack, declared that she saw a person of her acquaintance going to a chemist's shop, distant from the house, in a large flowing dressing-gown. This was her father; and M. Lallemand going out to inquire, found that the circumstance had occurred as she had related it.

"Being called in to give my opinion on the state of Madame N., I also observed many remarkable peculiarities. Although presenting a very healthy appearance, she had frequent *malaise*. The principal functions of the economy were performed with sufficient regularity—nutrition, for example, was perfect; but the appetite was fantastic to the extreme. A lively exaltation of the cutaneous sensibility was manifest in the cervical region, about the spinous process of the second vertebra. Even in the normal state, she complained much of the lightest touch there. All impressions which she received seemed to 'echo' there. Having once touched this part as gently as possible, Madame N. felt extreme pain there the whole day. The touch of a Napoleon caused less suffering; the temperature of the gold *rose rapidly* on the contact. This essay led me to try the application of a piece of magnetized steel. During one of the crises I touched the painful spot with this, and she manifested no uneasiness; on awaking, the ordinary suffering was less than usual; and these results have been repeated.

"After many attempts, I succeeded in establishing a communication by voice between us. Informed hastily by her husband that she was in a state of somnambulism, I went to see her. She was immovable in bed, and did not seem conscious of my arrival. My hand was placed in hers by her husband. Her limbs were in a complete state of relaxation, not presenting the rigid phenomena of catalepsy.

"At first Madame N. only repeated my last words, but at length seemed to recognise me, and said—'Ah! it is he who wishes to cure me; he has undertaken a difficult task; he wishes to give me sulphate of quinine; it is well, I have no objection; let him do what he will; besides, I know nothing; somnambulists generally say foolish things.' She then went on to describe the methods that had been before adopted for her cure, and mimicked with spirit the *brusquerie* of M Lallemand, and the gentle soothing manner of M. Planidoux.

"Before the application of the magnetized steel, I had once observed Madame N. during the sleep. She had announced during the previous crisis the day and hour when the next would occur; at this I proposed to be present. I was there before the time; the patient knew nothing of her own prediction; but a little before the time announced, she told me she felt inclined to sleep, and she lost gradually her accustomed vivacity. She became uneasy; the respiration became quicker, and slightly stertoreous; she then slept without the least agitation.

"At this latter examination, Madame N. had exacted from me a promise that I would not touch her—a promise which I should have kept, but during her husband's absence her arm was thrown out of bed, and struck violently a marble slab. I tried to replace it, but the attempt caused great suffering; and after she awoke, which took place without any consciousness of what had passed, she complained of great pain the whole length of the arm.

"Madame N. was occupied during her sleep with the same subjects that had formed the topics of her waking conversation. Thus, having been relating to me the death of a man whom she very much disliked, in her sleep she assured me 'que le diable lui-même ne voudrait pas de son âme.'"

"Case II. Madame B., a native of Havannah, æt. nineteen, married eighteen months before, had always before her marriage enjoyed good health, with the exception of some nervous pains with tendency to faintness. Nevertheless, she was occasionally affected with low spirits and involuntary tears. Sexual intercourse gave her some pain, and at the approach of the menstrual period she had considerable lumbar pains. All at once, at the beginning of Feb., 1841, without appreciable cause, she fell anew into repeated syncopal attacks, with hysteriform convulsions, delirium, and hallucinations. These symptoms, after some hours, were calmed by the use of assafœtida. On the following days, particularly after meals, there occurred slight crises, marked at first by impatience and irritability, then by insensibility of the organs of the senses, by uncertainty of action and torpor of the intellectual functions. Towards the middle of the month, the occurrence of the menstrual flow checked the recurrence of these phenomena; but a week had scarcely elapsed when the syncope, convulsions, lumbar pains, the delirium and hallucinations reappeared with increased frequency and violence. The first half of March was quiet;—on the 16th there were headache, vertigo, and pains in the groin. The patient said, also, that 'something ran along the eye, and the eyelids fell in spite of herself.' These phenomena again disappeared with the return of the menses. On the 26th, at midday, the convulsive movements returned; the ears tingled, the eyes closed; at last Madame B. neither saw nor heard, yet she spoke volubly, and expressed herself clearly. In this state she got up to go out, made two or three steps in the street, then suddenly complaining of feeling something go down from her head to her feet, she regained the use of her senses, and continued her walk. Similar attacks, generally preceded by renal pains, occurred frequently in the beginning of April. From the 12th to the 15th there occurred delirium and hallucinations, which left much weight of head behind. On the 23rd, Madame B. was frightened by a storm, and had syncope, convulsions, and delirium, which were repeated during three days. On the 26th, the accession assumed an entirely different form. To the preceding symptoms was added an excessive sensibility of the whole surface; the different parts of the body became successively motionless, and as if paralysed. She could not breathe without great labour, the tongue did not obey the will, deglutition was impossible, vision became extinct, and consciousness was lost. She was made to breathe assafœtida, and the symptoms subsided after twelve hours. In subsequent attacks there were other strange symptoms. There were, at first, convulsive movements; the globe of the eye rolled upwards so as only to show the sclerotica; there were hallucinations. She spoke incoherently; then her mind seemed to be enlightened, the ideas became connected, and her thoughts and language assumed an unwonted elevation. Although surrounded by friends, she thought herself alone. On awaking, she asked for food, and then slept peaceably until the next morning. Her features then were heavy; she recognised those about her, but knew nothing of what had passed the day before. There were repeated attacks of the same nature, during which she would recal events which had transpired in the previous crises. Sometimes when apparently well, and in all other respects collected, she would miscal her husband and all her friends. One of the attacks was complicated by an extremely violent lumbar pain. She said it felt as though each bone of the spine was divided from the other, and in each a boiling liquid.

"In September, being called upon again to take charge of Madame B., I had ample opportunities of verifying the singular phenomena of this affection, to which, also, many of our medical brethren can testify. The treatment which I prescribed was followed for some months by a notable amendment; but the weather having become very cold and wet, the crises occurred again at very short intervals. Their nature is shown in the following letter to me from her husband, an eminent *littérateur* and advocate. The clear and simple style of the document, and the honourable character of M. B., do not permit any doubt of the veracity of the narrative:—

"'The more I observe the strange malady of my poor wife, the more I am astonished at the phenomena of double existence which it produces in her. When she recovers her senses, she knows nothing of what has passed in the attack; when she is again ill, she recals the former attack with surprising fidelity. At these times the senses seem changed; she has even special ones; she hears certain words, and not others; she recognises the portraits of persons, and not the persons themselves. In what she does and says, all is connected and rational; she takes up the thread of ideas from one accession to another; orders the details of the *ménage*, and calculates her accounts without committing the least error. As the attacks succeed each other with great rapidity, she awakes doing things which she cannot explain—so little relation have the two lives to each other. Any pain, however slight, wherever originating, brings on these attacks; and their strength is proportionate to the pain. In its slightest degree, some attention is required to discover the symptoms of the nervous state; if this degree augments a little, she becomes animated, gesticulates and speaks with force, and her features are convulsed; if it be more advanced still, the condition of isolation is developed, she neither hears nor sees any one, speaks to herself, recites, runs, sings, laughs, and weeps; lastly, her discourse becomes incoherent, she confounds objects, loses certain senses, and supplies their place by others, which experience every day reveals to me. There are other effects not less *bizarre:* she is at such times deprived of the faculty of pronouncing diphthongs and certain consonants, seeming like a child learning to speak. Suddenly one of the senses fails, or an organ is paralysed: a finger, the eyelid, the tongue, the knee, the lips, &c. In other cases the exhaustion is so complete that all movement is impossible, yet she hears all that I say; for in coming out of this state, she repeats word for word what was said. Lastly, if on awaking she forgets all that has passed during sleep, it is not so with the sleep, in which she knows all in which she has been engaged before the accession of the affection.'

"During the numerous visits which I paid, I observed myself the greater part of the circumstances mentioned by M. B. Generally she did not recognise me, although she named me immediately on having shown to her a photographic portrait of me. She did not distinguish my voice, and to convince her of my presence I had to place under her eyes my signature, which she then compared with one which was underneath the above-mentioned portrait. I reproached her one day for ingratitude in not having answered some compliments which I had paid her; but as she did not seem to understand, I wrote it down. She then took a pen and excused herself, on the plea that she had not recognised me, but was very grateful for all my cares. Thus another anomaly was manifest, as she read this with eyes upturned under the lids, not having been able either to see or hear me. However this may be, the accidents became by degrees less frequent, and finally ceased. Since then Madame B. has become a mother, and she does not now retain any traces of her old sufferings."

*General Observations.*—It was the same with Madame N———. In both cases the affection was circumscribed within a period of four or six years.

In the last instance, so curious from the mobility and infinite variety of the symptoms, the characters of somnambulism were even more clearly marked than in the first instance. Not only could Madame B—— answer questions, and perceive objects otherwise than by sight, but she rose up, walked, rode, eat, looked after the house, wrote, made up her accounts—did, in a word, all that a somnambulist could do. The attack being passed, she forgot every act connected with it, but on the next occasion took up the broken thread. Nevertheless, if in all these particulars these two cases seem allied to natural somnambulism, in others, not less important, they differ widely from it, and require to be placed in a separate category. Whilst natural somnambulism occurs without shock, in the night, in course of sleep, these affections come on indifferently at any hour of the day, when the patient is awake, and under the form of a nervous crisis. Lucid sleep is not then a simple modification of ordinary sleep; it results from the violence of the cerebral spasm (*du spasme cérébral*), and very often the attacks are ushered in by divers precursory phenomena—general disquiet and uneasiness in the limbs, headache, vertigo, sadness, weeping, desire to laugh involuntarily, yawning, numbness, syncope, agitations or convulsions. In one of our cases there was pain in the nape of the neck; in the other, lumbar pain and general exalted cutaneous sensibility. (M. Ferrus proceeds to indicate the further differences between this condition and that of natural somnambulism, which may be seen by comparing the phenomena of the cases recited.)

Considering these numerous differences, we are naturally led to admit two sorts of somnambulism; one equally known to physicians and the public, and another which is related to certain periodic neuroses, if even it may not be considered a variety of these, upon which the phenomena of somnambulism are engrafted; these occurring always at a comparatively advanced period of the affection, the earlier stages of which resemble a *mélange* of syncope, catalepsy, hysteria, and delirium.

The astonishment and the lively interest and curiosity which attach to the singular symptoms of this kind of somnambulism, have caused authors to neglect the medical and practical view of the question; and so we know but little of the etiology, the progress, and prognosis of this affection. We may conjecture that the general causes are those common to nervous maladies. As to the immediate causes, we are still in the dark. In these two cases, menstruation exercised an opposite influence. In one, the crises corresponded with the approach of the period; in the other, they were temporarily relieved by it. In the course of the malady, the general health and intelligence were but little affected, and the sequelæ were unimportant. Yet we may suppose that from these, as from other neuroses, might result mental alteration, profound and durable. In neither case have we traced any direct hereditary influence. Both were born and brought up in hot climates; both were cured after four or five years of suffering; both became pregnant after some years of marriage, and after the entire cessation of the affection. Their children are living. Both have become widows, without any recurrence of the attacks from the profound grief which they experienced. The question arises as to the relation of such phenomena to those of mesmerism: we only propose, but do not at present attempt to answer it. One word in conclusion: we must admit nothing without examination—without proofs and prudent investigation. This obligation is imperious to every serious inquirer: it is equally the duty of every friend of science to repudiate nothing which experience may render manifest. In a word, we must neither be in haste to recognise the existence of extraordinary facts, and to deduce theories from them, nor irrevocably to reject phenomena, because they pass the ordinary limits of our knowledge.

---

*On Disordered Sentiments and Affections.* By M. Auzony.

In the "Annales Medico-Psychologiques" for January, 1858, M. Auzony gives an analysis of 415 cases of insanity observed during three months at the Asylum at Fains, with a view to ascertaining the frequency and conditions of the complications of intellectual disorders with those of the affections and emotions. For this limitation of time, certain reasons are given which lead the author to prefer a short observation to a long one.

The emotions may be directed singly or simultaneously to four objects—God, the individual himself, his family, and his species,—and are manifested as

1. Adoration, honour, worship, love, fear, &c.
2. Instinct of self-preservation.
3. Instinct of reproduction, including the sexual emotion, conjugal and parental affection, and the general feeling of the ties of consanguinity, &c.
4. The instinct of social relations, including politeness, benevolence, pity, esteem, gratitude, justice, generosity, admiration, courage, enthusiasm, patriotism, &c.

All these are susceptible of exaggeration, of diminution, of perversion, and of abolition. Thus, in the first class, *exaggeration* produces *mysticism*, *superstition*, and *demonomania*; *diminution* produces *scepticism*, *incredulity*, *Voltaireianism*; *perversion* produces *blasphemy*, and *apostacy*; and *abolition* produces *indifference*, *materialism*, and *atheism*. The corresponding results in the second class are, from *exaggeration*, egotism, covetousness, fear, pride, ambition; from *diminution*, prodigality, rashness, carelessness; from *perversion*, avarice, intemperance, mortifications, voluntary mutilations, and suicides; from *abolition*, apathy and inertia. In the other classes, similar perversions result in hatred, treason, jealousy, erotism, contempt, mockery, harshness, pride, ingratitude, revenge, cowardice, homicide, &c.

The kind of malady in its successive phases influences greatly the mode of alteration of these emotional faculties. Thus, in paralytic insanity, there is most frequently observed at the commencement an exaggeration of the sentiments, then perversion, followed by enfeeblement and obliteration; and similar variations are observed in the other forms. From the tables given it appears that "delirium of the affections" almost constantly complicates that of the intellect. Out of the four hundred and fifteen cases, there were only thirty in which the *integrity* of the emotional faculties was preserved; and it cannot be doubted that a longer observation of these cases would have greatly reduced the proportion. Of these thirty, eight were imbecile; seven were cases of intermittent mania; six were melancholia; four were monomania; two were mania; two were epileptics; and one was paralytic insanity. In the three remaining sections of the classification adopted—stupidity, dementia, and idiocy—all the cases were complicated with some emotional or *affective* disorder.

The classification here alluded to is in itself not without interest. The 415 cases comprised all that were treated during the three months at Fains, and are divided into two groups in some degree corresponding* to acute and chronic cases. The first includes mania (36); intermittent, or remittent mania (53); monomania (43); lypemania or melancholia (73); in all 205 cases. The second includes stupidity (4); dementia (71); paralytic insanity (14); epileptic insanity (27); imbecility (71); and idiocy (23); in all 210 cases.

These two groups, nearly equal in number, vary much in their relations to emotional complications. In the former group, the emotions were perverted or exaggerated in 30 and 34 per cent. of the cases; whilst in the latter group the proportions were but 10 per cent. of perversions, and 4 per cent. of exaggerations. On the other hand, in the former group, there was only a proportion of 20 per cent. of enfeeblement, and six per cent. of abolition of these faculties, whilst in the latter, 35 per cent. were enfeebled, and 45 per cent. abolished.

Of the whole number (415), in 85 cases (or 20 per cent.), the sentiments were perverted; in 77 cases they were exaggerated; in 116 (or 28 per cent.) they were enfeebled; and in 109 (or 26 per cent.) they were abolished.

The perversions of affective sentiment were by far the most frequent in lypemania, occurring in 27 cases out of 73. The exaggerations were proportionally the most frequent in mania, occurring in 19 out of 53 cases of the remittent character, and in 15 out of 36 of the continued kind. Diminution of the affections was observed by much the most frequently in imbecility, viz.—in 34 out of 71 cases. Abolition was, as might be expected, most frequent in dementia—in 44 out of 71 cases. The following table will facilitate reference:—

| State of Emotional Faculties. | | 1st Group. | 2nd Group. | Total. |
|---|---|---|---|---|
| Integrity | in | 19 | 11 | 30 |
| Perversion | „ | 63 | 22 | 85 |
| Exaggeration | „ | 69 | 8 | 77 |
| Diminution | „ | 42 | 74 | 116 |
| Abolition | „ | 12 | 95 | 107 |
| Whole number | | 205 | 210 | 415 |

M. Auzony remarks, in conclusion, that although as a general rule the disorder of the affections is consequent upon, and due to, that of

* Not M. Auzony's analogy, we ought to add.

the intellect, the order may be reversed, and the "effect become the cause." Thus, a young advocate of perfectly sound mind, and with no hereditary tendency to insanity, having obtained the promise of the hand of a young lady to whom he was devotedly attached, could not support the happiness, and became a prey to incurable melancholy, with hallucinations and constant chimerical terrors. He adds that this "delirium of the affections" requires the same kind of treatment as that of the intellect; but that the moral treatment ought to "predominate, and, in fact, be almost exclusive. It requires, also, on the part of the medical attendants, an increase of vigilance, of tact, and of discernment."

---

### *Case of Mania with Homicidal Tendency.* By M. Morel. (Abridged.)

On May 3rd, 1852, Joseph Chanel, a road-labourer in the department of the Vosges, met an acquaintance named Olivier, whom he passed apparently sulkily, without taking any notice of his salutation. Immediately afterwards Olivier heard piercing cries, and on turning saw Chanel pursuing a child, whom he seized by the collar, and killed by repeated blows with a hatchet. Olivier wished to advance to prevent the murder, but dare not, as Chanel, brandishing his weapon, threatened to kill him also if he came near. He passed several men whom he also threatened to kill, "as he had done that other one," if they molested him. Returning home quietly, after purchasing his provisions, he was arrested by force of numbers, and said, "What do you want? I have killed a child, I don't repent of it; it is time to have done with them." Examined, he acknowledged the crime without any expression of regret; he wished to kill some one on that day, and had taken his hatchet out on purpose. Taken to the prison of Epinal, he was violent, and said that "all his food was poisoned." The physician, however, did not think him insane. On the 23rd of May, he was removed to the Asylum of Maréville, to be under the observation of MM. Morel and Blondlot.

The report of these alienists is divided into three parts:—

1. His present mental condition.
2. His antecedents.
3. The conclusions drawn from these.

1. On his admission to Maréville, Chanel was violent, and abused and attempted to strike the officials. His expression was sombre, his eye threatening; he required the strait waistcoat. He answered contemptuously or abusively all questions and observations: "What are you preaching about? I don't like sermons." He whistled and sung, but ultimately answered more calmly. "What I have done, is done—it was time to end it; if you had suffered as I have you would soon see. . . . ." All that could be understood of his sufferings was that all his food was constantly poisoned by some unknown agencies, which he called "Magogie" and "Question." Moreover, he affirmed that he was not insane, but expressed the utmost indifference to his fate.

There was no sign of physical disturbance; pulse about fifty; tongue clean and natural; appetite normal; sleep apparently untroubled. Sometimes he was mute, but after the douche he became more tractable.

On the 27th there appeared a change; he was no longer violent, but depressed and looked on the ground constantly. On being questioned, he would only answer "You know all about it."

Interrogated on the 3rd of June, he answered as usual; but M. Blondlot attempted to reason with him: "Granting that your food was poisoned, is that a reason for killing a child?" "That may be, but it was time to end it—as well him as another." On the 15th he again became violent, and refused food. There was a furred tongue, which he attributed to poisoned food as usual. An emetic, which acted violently and copiously on the stomach and bowels, and a bath, restored the former condition. When again tranquil, Dr. Morel urged him to write to his mother, "as he was likely to be condemned to death." He did so, but with indifference as to his fate. On the 5th of July, renewed violence with public indecency, attended as before by digestive disorder. M. Morel remarks that there was a striking periodicity in these functional perturbations; and that they were always attended by greater mental agitation. Up to the 26th of September nothing more could be elicited by question or observation, than that his food was poisoned, not by a poison that kills, but that causes great discomfort. His vengeance must fall upon some one, no matter whom; and he had killed the child to make an end of the business (*pour que cela finisse*). He was ready to do the like again if tormented any more. He was not insane, and would rather die on the block than be thought so. For the rest, total and brutish indifference.

2. At the age of nineteen he enlisted voluntarily, and after seven years' service he was discharged with a certificate of good conduct, in April, 1838. In 1848 he was local commandant of the National Guard, and in the same year was engaged to be married; but he committed certain extravagances, for which he was removed to the Asylum of Maréville, as likely to compromise the public safety. He was there quiet and orderly, and in a few months was restored to his family and his public functions. Before this it appears he had taken to drinking, had complained of headache, and had cranial and facial erysipelas, with epistaxis and bleeding from the ears. After this, his gait was occasionally hesitating, and his face frequently injected; his arms were affected with convulsive actions also. It was soon after this that he entered Maréville. Leaving the asylum in February, 1849, he was observed in a few months to be affected with great religious exaltation of sentiment. He attended all religious services, fasted rigorously, and confessed such sins that he said the priest was *lâche* for giving him absolution.

After the midnight mass, in 1851, he again changed completely—believed in nothing, blasphemed much, committed great excesses, and wrote threatening letters to the priests and civil authorities. Then appeared for the first time his dominant idea of the poisoned food, on which subject he quarrelled with all his family, and, becoming gradually more and more violent, at last committed the crime related.

3. In the judgment passed on this case, M. Morel reviews the preceding circumstances, weighing their value and significance, and concludes that Chanel is a "depraved (*abruti*) being, in whom the sentiments are completely disorganized, and whose intellect has never been well developed. We have carefully guarded (he continues) against a simulated insanity; we have observed him at all hours of the day and night; we have tried to ascertain if these periodical returns are the expression of a pathological condition: we have continued our observations for four months, and we are convinced that all the acts of this man were the result of a general perturbation of the intellectual faculties. Chanel is a hypochondriacal maniac, with systematic ideas of persecution and evil influence exercised upon his person. This affection presents periods of exacerbation, and whatever may be the decision of the law, we are of opinion that any return to society should be interdicted to this individual, perverted both in his intelligence and his emotions."

In commenting on this case, M. Morel takes occasion to contest the theory of monomania as set forth by Ruel, Esquirol, Marc, and Georget. He rejects incendiary, homicidal, and other monomanias, as forming distinct affections of themselves, and considers them as "tendencies and symptoms" of the principal fundamental malady, general mental derangement. In support of this he analyses certain cases brought forward as illustrative of these monomanias, and shows that in the history of such cases there was ample reason to perceive that, independent of the crowning acts which gave character to the disease, there was general derangement of the mental functions, more or less explicitly marked.

---

### *On the Pathogenic Influence of Loss of Sleep.*
### By M. E. Renaudin.

M. Renaudin's observations on this subject are so interesting and important, not only as showing the powerful influence of *insomnia* in the production of disease, but also as indicating the loose analysis of phenomena which is too often made in the science of etiology, that we shall endeavour in as brief a space as practicable to give the whole of his views.

"In tracing back effects to causes, we are often content with having found a cause which *may have* produced the effect, without carefully examining the whole of the phenomena; we often arrive at etiological data accepted without control, transmitted without examination, and by and by transformed into axioms which no one thinks of contesting. Where is the young girl who has not indulged in one or two dreams of love? If insanity follows some such dream, the statistics of moral causes are increased by a unit. If it follows a deception or a loss of property, again this is accepted without inquiry as the 'moral cause' of the affection. Lately the list of causes has been increased by another—viz., the residence amongst the insane. But between the fact which we consider the cause, and the malady which we regard as the effect, there are intermediate events which we pass over in silence; which nevertheless often contain the pathogenic knot which it is of so much importance to unloose. I confine myself on this occasion to speak of one only, which plays an important part in the production of disease—loss of sleep.

"Ordinary maladies exhibit notable modifications, according as they are complicated or not with insomnia. Very powerful is the influence of sleep over nervous and inflammatory diseases, where opiates produce remarkable results, as well as over affections of the digestive organs. We may observe daily how prejudicial is interrupted sleep to the performance of the digestive functions. Derangement is produced when the normal duration of sleep is abridged by mental excitement or occupation. This effect varies from age to age, and the younger the subject the more necessary is sleep. If too often interrupted, there results a state of cerebral excitement, which prevents the possibility of the return of natural sleep without the employment of therapeutic agency. The confusion of ideas which is constantly experienced in the transition from the sleeping to the waking condition, becomes constant in the cases alluded to, and is aggravated into a form of insanity, the special type of which depends upon other causes which have preceded or induced this sleeplessness. The citation of certain facts will illustrate the position.

"When, in 1842, I undertook the direction of the Asylum of Fains, the cellular system was in great repute, and the thirty apartments which were in each section contained in a very small space individuals who could give themselves up to all the vagaries of their riotous delirium. The walls were thin, and yell arousing yell, there resulted an insomnia concert, which aggravated and perpetuated the delirium. The system being changed, this agitation disappeared; and amongst the patients many owe their recovery to the peace and quiet they were thus enabled to enjoy. But before this took place I had been taught a very important lesson. The nurses attached to this quarter began very soon after their admission to lose those qualifications which had induced us to select them. Their character became awkward, their irritability increased day by day, their intelligence declined gradually, and in some impending stupidity rendered their dismissal necessary, as well as a brutality utterly different from their primitive character. The privation and interruption of sleep had caused these changes, which disappeared quickly when they were removed and could have rest and quiet. The same results accrue to the nurses who sleep in quiet wards, and yet are unable to rest without interruption from fear.

"On my entrance at Maréville, I found the same results attending the cellular system. Whilst this was in operation the nurses had to be almost constantly changed, from causes similar to those just mentioned; and very few were found who could resist the consequences of such deprivation of sleep—consequences in many cases very serious.

"A young nurse was admitted a short time ago into the asylum, when, for some nights, the turbulence of the patients in her division interrupted constantly her sleep. Not daring to acknowledge her fatigue and claim some hours of repose, she laboured on without complaining of any inconvenience. Four days passed thus, but on the fifth she presented all the characters of an access of mania, hallucinations, excitement, restlessness, incoherence, &c. Had this continued long, the mania would have become fixed, but the cause being recognised, she soon recovered by opiates and repose. Two other cases more obstinate occurred within a short time. These facts are certainly exceptions, but the statement of such extreme cases will throw light upon other slighter phenomena.

"A man of good constitution, of jovial temper, and most irreproachable character, was suddenly informed that a grave accusation had been calumniously brought against him, and the letter in which it was contained was shown him. The blow was too severe, and he exhibited an excitement almost maniacal. The first emotion passed away, he resumed his occupations, and his family hoped that all was well. They reckoned, however, without taking into consideration the insomnia, which remained as the final consequence of the moral tortures he had undergone. The least event startled him, the circulation was accelerated, the beatings of the heart became tumultuous, and after an incubation of some days, a violent attack of mania broke out. This was attributed *directly* to the moral cause, without investigating the process of evolution, and for some time nothing was done but to combat congestion, &c. At last opium and digitalis were given with notable good results; but for some reason or other this medication was abandoned, and the mania manifested itself anew with great violence, and will now doubtless be incurable. This case indicates the necessity of seizing *opportunity* as well as the proper remedies, or the time comes when treating properly the original cause will no longer remove the effect; the habit has become chronic. Sleep, re-established too late, fails to bring back calmness or the reaction of reason.

"I have been able in another case to trace the pathogenic influence of want of sleep in the production of delirium. A young girl, arrived at the age when the *besoin d'aimer* is strong, had some hysterical symptoms, which ended in a maniacal attack, which was most violent when she saw any youth with a resemblance to an ideal formed in her own mind, during a state of hallucination consequent upon loss of sleep. Her friends at first saw only the agitation, without seeking deeply into the cause, and instead of trying to obtain sleep they made her take prolonged baths at Plombières; the consequence of which was that the insomnia became chronic. The use of opiates produced a notable amelioration; when she could sleep the hallucinations almost disappeared, but as soon as the insomnia returned, the delicious conceptions returned also, and became in some measure fixed.

"I add the relation of another case which strongly indicates the necessity of examining minutely into the initial phenomena of this formidable malady:—It is that of a lady in whose family it was impossible to trace the least hereditary taint, herself being a person of extraordinary force of character. Her husband lost a large amount of money, and shortly afterwards she fell into a state of lypemania, against which the resources of medical skill have been hitherto powerless. Grief was regarded by all as the cause of this, but a more minute inquiry revealed a very different kind of sequence. These are the facts: one day she entered the house of a relative who had committed suicide,* and saw the body without having been forewarned. Wishing to relieve her husband of some care, she examined the papers of the deceased, and found details of the most delirious conceptions. She destroyed every trace of this, and had scarcely finished when her husband entered, and informed her of his losses. She appeared at first soothed by the consolations of her friends, but *continued sleepless;* no one inquired about this fact, and she did not think of mentioning it. A few hours of slumber might have saved her, but prolonged insomnia was followed by profound stupor, which will most probably terminate in dementia."

Loss of sleep seems also to be the primordial element of that marasmus which terminates the life of certain maniacs, who seem to have no other lesion than a gradual loss of power, a true inanition from default of assimilation. We also remark the comparative harmlessness of even great excitement, when sleep is not interfered with; and the dangers of the period of prostration are increased in proportion as the

* The ensuing relation seems scarcely consistent with the previous statement as to the absence of hereditary tendency.

period of exitement has been marked by more or less insomnia. It is ordinarily by insomnia that the periodic returns of mania commence, followed by gastric disorder; and if we carefully observe these symptoms in those subject to periodical attacks, we may not infrequently cause the attack to abort, or at least make it a very mild one. Again we observe in continued mania, that insomnia marks the periods of aggravation and *recrudescence* of the delirious conceptions. In many cases, so long as regular sleep can be obtained, the malady is reduced to a quiescent state, a sort of "abstract virtuality;" but a few days of insomnia suffice to light it up actively again. One of our monomaniacs is in this case; he is the creator of heaven and earth; but beyond this conception he is one of the most polite and mild of men; but deprived of sleep from any cause, he becomes irritable, and a prey to extreme excitement; and thus his theoretic delirium becomes open and practical.

Chronic insomnia is frequent amongst those who pass lonely lives, especially females; this arises from nocturnal terrors, and is often the cause of insanity. There is, at this time, in Maréville, a woman aged 40, who, under the influence of these nocturnal terrors, has become gradually subject to hallucinations of the eye and ear, which at first ceased when she was no longer alone. After some time the *expressions* of her terror ceased, and an amendment was anticipated; but instead of this, it was found that the supposed phantom had acquired more force and power, even sufficient to repress all manifestation of affright, by the order of an audible voice. This person would not have arrived at this point without the insomnia and the solitary dwelling.

"In general, when a moral cause has been the point of departure of mental alienation, it is rare that insomnia has not had an important share in the development of the affection, which, prepared by the psychical element, is only definitively organized when the somatic element has done its work by loss of repose."

---

